# Severe hyponatremia in a patient with schizophrenia associated with prolonged consciousness disturbance

**DOI:** 10.1007/s13730-016-0234-1

**Published:** 2016-10-18

**Authors:** Kunihiko Yasuda, Takatsugu Iwashita, Yosuke Tayama, Yuko Makino, Ayumu Watanabe, Tatsuro Sano, Taisuke Shimizu, Tomonari Ogawa, Koichi Kanozawa, Hajime Hasegawa

**Affiliations:** 1 Department of Nephrology and Hypertension, Faculty of Blood Purification, Saitama Medical Center, Saitama Medical University, 1981 Kamoda, Kawagoe, Saitama, 350-8550 Japan; 2 Department of Radiology, Saitama Medical Center, Saitama Medical University, 1981 Kamoda, Kawagoe, Saitama, 350-8550 Japan

**Keywords:** Hyponatremia, Syndrome of inappropriate secretion of antidiuretic hormone, Osmotic demyelination syndrome, Neuroleptic malignant syndrome

## Abstract

Hyponatremia presents with various central nervous system symptoms during its course and treatment. We treated a patient who presented with a prolonged consciousness disorder and was suspected of having complications of neuroleptic malignant syndrome and osmotic demyelination syndrome (ODS) during the treatment for his hyponatremia, which was caused by syndrome of inappropriate secretion of antidiuretic hormone (SIADH). The patient was a 30-year-old Japanese man who had been under treatment for schizophrenia. He presented with profound hyponatremia (96 mEq/L) and a consciousness disorder. Because he was taking a number of antipsychotic drugs and since psychogenic polydipsia was present along with laboratory findings, the patient was diagnosed with SIADH. However, the consciousness disorder reappeared after his serum sodium concentrations were normalized, and it persisted over a long period. Although ODS was suspected from the clinical course and imaging findings, there were several inconsistencies, such as the lack of quadriplegia. The patient also showed muscular rigidity and fever, and we, therefore, diagnosed complications of malignant hyperthermia syndrome caused by the discontinuation of all antipsychotic drugs at the time of onset. There have been no reports of complications of these two conditions, and we report this case for its clinically valuable information.

## Introduction

Severe hyponatremia is often associated with a variety of central nervous system symptoms, including disturbed consciousness. An appropriate assessment of hyponatremia is necessary before the most suitable treatment can be administered. Hyponatremia is generally classified as either dilutional or salt-losing hyponatremia, with differing therapeutic approaches. The proper differential diagnosis is thus essential when choosing a therapeutic strategy.

We recently treated a patient with both severe hyponatremia and schizophrenia who had been prescribed multiple antipsychotic agents. The incidence of hyponatremia is higher in patients with an underlying psychiatric disorder [1, 2], and the involvement of psychotic polydipsia and the resetting of antidiuretic hormone (ADH) regulation are suggested. We presume that similar mechanisms may be involved in the cause of hyponatremia in our patient’s case. Syndrome of inappropriate secretion of ADH (SIADH), characterized by a disorder of free water excretion, typically presents with dilutional hyponatremia. SIADH cases associated with drug use are frequently experienced in clinical settings.

In addition, central pontine myelinolysis (CPM) is a well-known nervous system abnormality observed in patients during their recovery from hyponatremia. In the present case, we observed a variety of neurological signs during the clinical course, including a persistent disturbance of consciousness. Our analysis of this patient’s case provides valuable clinical information regarding the electrolyte imbalance as well as the neurological symptoms that may be observed during the course of treatment.

## Case report

The patient was a 30-year-old unemployed Japanese male. He was diagnosed with schizophrenia at the age of 23 and had been treated at a psychiatric hospital on an outpatient basis. He exhibited water intake behaviors, and he had been drinking approx. 10 L/day for the past several years. At 29 years of age, the patient showed mild disorientation and was hospitalized for water intoxication; the details are unclear. From 6 days before he was admitted to our hospital, he became aware of nausea that gradually worsened. Two days before his admission, in the morning, he showed mild disorientation and a speech disturbance, and an ambulance was requested for transportation to a regional emergency hospital. There was no history of illnesses or accidents, such as head injury, epileptic seizures, or severe infections. No obvious hereditary diseases were observed.

During the patient’s transfer the regional emergency hospital, his Glasgow Come Scale (GCS) was E4V1M5, but there were no obvious abnormal findings in the head and neck, chest, or abdomen. Physical findings did not suggest either overhydration or dehydration. Moreover, no obvious neurological abnormality was observed such as the deep tendon reflex abnormalities or presence of pathological reflexes. A chest X-ray at the time of admission showed no pleural effusions, cardiomegaly, or pulmonary congestion.

The patient had been treated for schizophrenia with more than 10 different types of oral medication (Table 1). Since these drugs are known to cause drug-induced syndrome of inappropriate secretion of ADH (SIADH), they were discontinued at the time of admission to the emergency hospital. Table 2 shows the results of the blood and urine tests performed at the time of admission to the emergency hospital. The patient was treated for severe hyponatremia (96 mEq/L) and hypokalemia (1.9 mEq/L), and was transferred to our hospital on day 3 of his stay in the emergency hospital, so that he could receive further specialized treatment.

**Table 1:** Preadministered antipsychotic medications

	Administered dose	Regular dose in Japan
SIADH-inducible (reported) medications		
Chlorpromazine hydrochloride	225 mg	30–100 mg
Levomepromazine	50 mg	25–200 mg
Phenotiazine derivatives	1 tablet	1–2 tablets
Risperidone	8 mg	12 mg
Sodium valproate	1000 mg	400–1200 mg
SIADH-uninducible medications		
Biperiden	4 mg	3–6 mg
Trihexyphenidyl hydrochloride	6 mg	6–10 mg
Alprazolam	1.2 mg	1.2 mg
Estazolam	2 mg	1–4 mg
Flunitrazepam	2 mg	0.5–2 mg
Quazepam	15 mg	20 mg

**Table 2:** Laboratory test of blood and urine

Urinalysis		Endocrinology	
Urine specific gravity	1.011	Adrenocorticotropic hormone (pg/mL)	160.3
Red blood cells (cells/HPF)	1–4	Free T4 (pg/mL)	1.8
White blood cells (cells/HPF)	0–1	Free T3 (pg/mL)	2.2
pH	5.5	Thyroid stimulating hormone (μIU/mL)	0.2
Protein	+1	Cortisol (µg/dL)	37.3
Sugar	Negative	Aldosterone (pg/mL)	52.7
Ketone	Negative	Plasma renin activity (ng/ml/h)	6.9
Urine chemistry		Blood cell count	
Creatinine (mg/dL)	70	White blood cells (/µL)	13,100
Urea nitrogen (mg/dL)	271	Hemoglobin (g/dL)	13.8
Na (mEq/L)	53	Platelet (/µL)	122 × 10^3^
Cl (mEq/L)	17	Blood gas analysis	
K (mEq/L)	37.5	pH	7.52
Urine osmolality (mOsm/kg)	332	pCO_2_ (mmHg)	50.0
Blood biochemistry and immunology		pO_2_ (mmHg)	63.5
Na (mEq/L)	96	HCO_3_^−^ (mmol/L)	40.2
Cl (mEq/L)	46		
K (mEq/L)	1.9		
Corrected calcium (mg/dL)	8.4		
Total protein (g/dL)	6.9		
Blood urea nitrogen (mg/dL)	9.8		
Creatinine (mg/dL)	0.90		
Estimated GFR (mL/min)	82.0		
Aspartate transaminase (IU/L)	30		
Alanine aminotransferase (IU/L)	16		
Lactate dehydrogenase (IU/L)	266		
Uric acid (mg/dL)	8.6		
CRP	0.4		
CK	574		
Plasma osmolality (mOsm/kg)	195		

After he was transferred, we performed an echocardiogram to assess the body fluid volume, which revealed a left ventricular diastolic dimension of 48.4 mm, and an ejection fraction of 64 %. We, therefore, estimated that there were neither major increases nor decreases in the extracellular fluid (ECF) volume. In addition, since the other clinical findings showed no evidence of increases or decreases in ECF, and no excessive activity of the renin-aldosterone system was observed, we considered the patient euvolemic. The fractional Na excretion was 0.45 % and was considered appropriate for an estimated glomerular filtration rate (eGFR) of 82 mL/min.

The cation ratio in the urine and blood [(urine Na + urine K)/serum Na] was <1.0, and the values for free water clearance were negative, and we thus concluded that the disturbance of free water excretion was the pathological basis of this case. We speculated that this was likely to have been caused by drug-induced SIADH, as the patient had been taking a large number of oral antipsychotic agents, and there were no intracranial or intrathoracic abnormalities. We also noted that the patient had psychotic polydipsia due to the psychiatric disorder, and we speculated that this also had an exacerbating effect on the hyponatremia.

During the patient’s stay at the emergency hospital, his water consumption was restricted to ≤500 mL/day, and because the hyponatremia was associated with a consciousness disorder, he was administered 3 % hypertonic saline and an infusion fluid mimicking the extracellular fluid. His serum Na and urine Na concentrations were monitored every 4 h, and the replacement speed was adjusted accordingly. At 12 h after the beginning of electrolyte correction, the administration of hypertonic saline was discontinued, because the elevation of Na concentration was 12 mEq/L at that time. On the day of transfer to our hospital, the patient’s serum Na and K concentrations had already improved to 121 and 2.1 mEq/L, respectively. The consciousness disorder had improved to a GCS of E4V4M6, by the time, the patient was transferred to our hospital as the serum Na concentration increased, and conversation with the patient became possible.

**Fig. 1:**
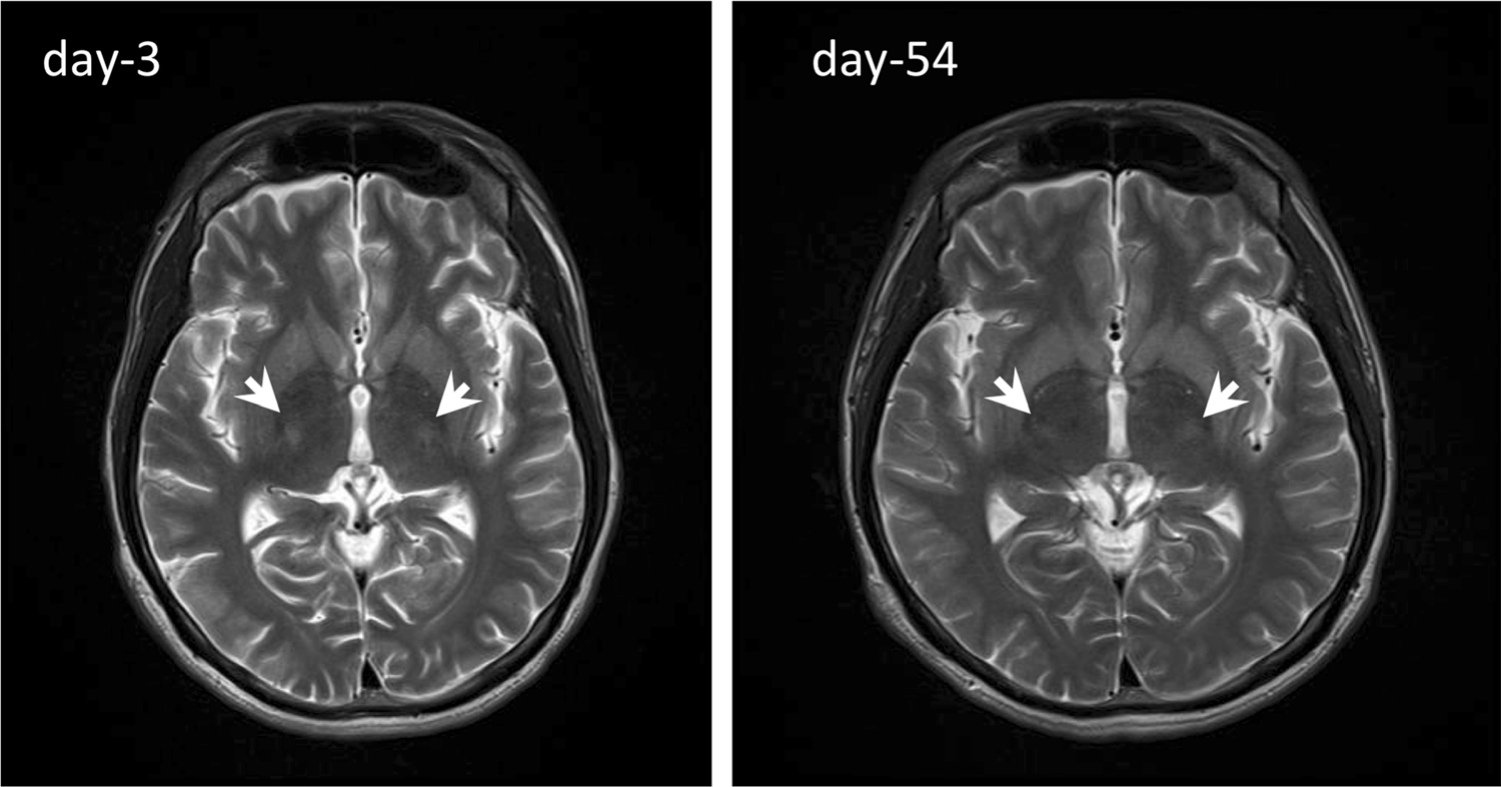
Brain MRI study (T2-weighted image) on hospital day-5 and day-54. *White arrowheads* indicate high-intensity signals in the ventral posterolateral nucleus (VPL)

However, from hospital day-2, the patient’s state of consciousness deteriorated (GCS E4V1M4) and conversation became impossible again; no obvious paralysis was confirmed. A brain MRI was performed on hospital day-3 to make a differential diagnosis, and a high-intensity signal in the ventral posterolateral nucleus (VPL) was observed on T2-weighted images (Fig. 1). At this point, we suspected a condition resembling extrapontine myelinolysis (EPM) on the basis of the speed of the serum Na correction and the brain MRI findings. Thereafter, the patient’s serum Na concentration normalized, but no change was observed in his state of consciousness.

Starting on hospital day-6, the patient developed a fever of ≤38 °C and muscle stiffness associated with elevated creatine kinase values. Muscle stiffness and excessive sweating were observed on hospital day-8. However, the C-reactive protein (CRP) value was approx. 0.4 mg/dL, and no infectious disease symptoms were observed. All oral antipsychotic agents were discontinued when the patient was admitted to the emergency hospital. Taking this into consideration, we considered neuroleptic malignant syndrome as a differential diagnosis, and we thus performed administering benzodiazepines.

On hospital day-10, we also considered the possibility of exacerbated schizophrenia, and an electroencephalogram (EEG) was obtained. The EEG showed diffuse slow waves, and the level of brain activity was thus considered low. Accordingly, we suspected that the patient’s consciousness disorder was caused not by an exacerbation of the psychiatric disorder, but rather by some functional cerebral disorder. After hospital day-19, the patient’s state of consciousness improved, and he regained the ability to speak. His muscle stiffness and sweating were also reduced.

On hospital day-29, a repeat EEG showed decreases in diffuse slow wave activities, and a moderate amount of α-waves was observed. It appeared that this reflected an improvement in the patient’s state of consciousness. However, brain MRIs performed on hospital day-17, day-36, and day-54 demonstrated persistent findings of a high-intensity signal in bilateral VPL, and this was not consistent with the changes in the state of consciousness (Fig. 1). Thereafter, the patient became ambulant on hospital day-48. He was transferred to the rehabilitation department on hospital day-78, and was discharged on hospital day-91.

## Discussion

We treated a patient with severe hyponatremia who had been taking several oral medications for a psychiatric disorder. A persistent consciousness disorder appeared after the patient’s serum Na concentration was corrected, and it was clinically difficult to differentiate between neuroleptic malignant syndrome and osmotic demyelination syndrome.

On the basis of the clinical findings, it appeared that a clear increase or decrease in extracellular fluid was unlikely to have occurred. In addition, we did not observe any findings indicating excessive activity of the renin–angiotensin system, and it was thus determined that the patient was generally in a euvolemic state. No increase was observed in the urinary Na excretion rate, eliminating the possibility of Na-losing hyponatremia. Despite the patient’s severe hyponatremia, the urine osmolarity was greater than the plasma osmolarity, and the urinary to plasma cation ratio was also >1.0. The free water clearance also showed a negative value. It thus appeared that the patient was in a state of free water reabsorption at the time of admission to the emergency hospital.

Based on the above findings, we classified this patient’s hyponatremia as dilutional hyponatremia caused by a free water excretion disorder, and we suspected SIADH. At the time of the patient’s admission, the supply of ADH test kits was temporarily suspended in Japan, and thus, ADH concentrations could not be measured throughout the country. Consequently, we were not able to measure the patient’s ADH concentration, but on the basis of the above-described laboratory and clinical findings, we believed that our clinical diagnosis of SIADH was likely to be correct.

**Fig. 2:**
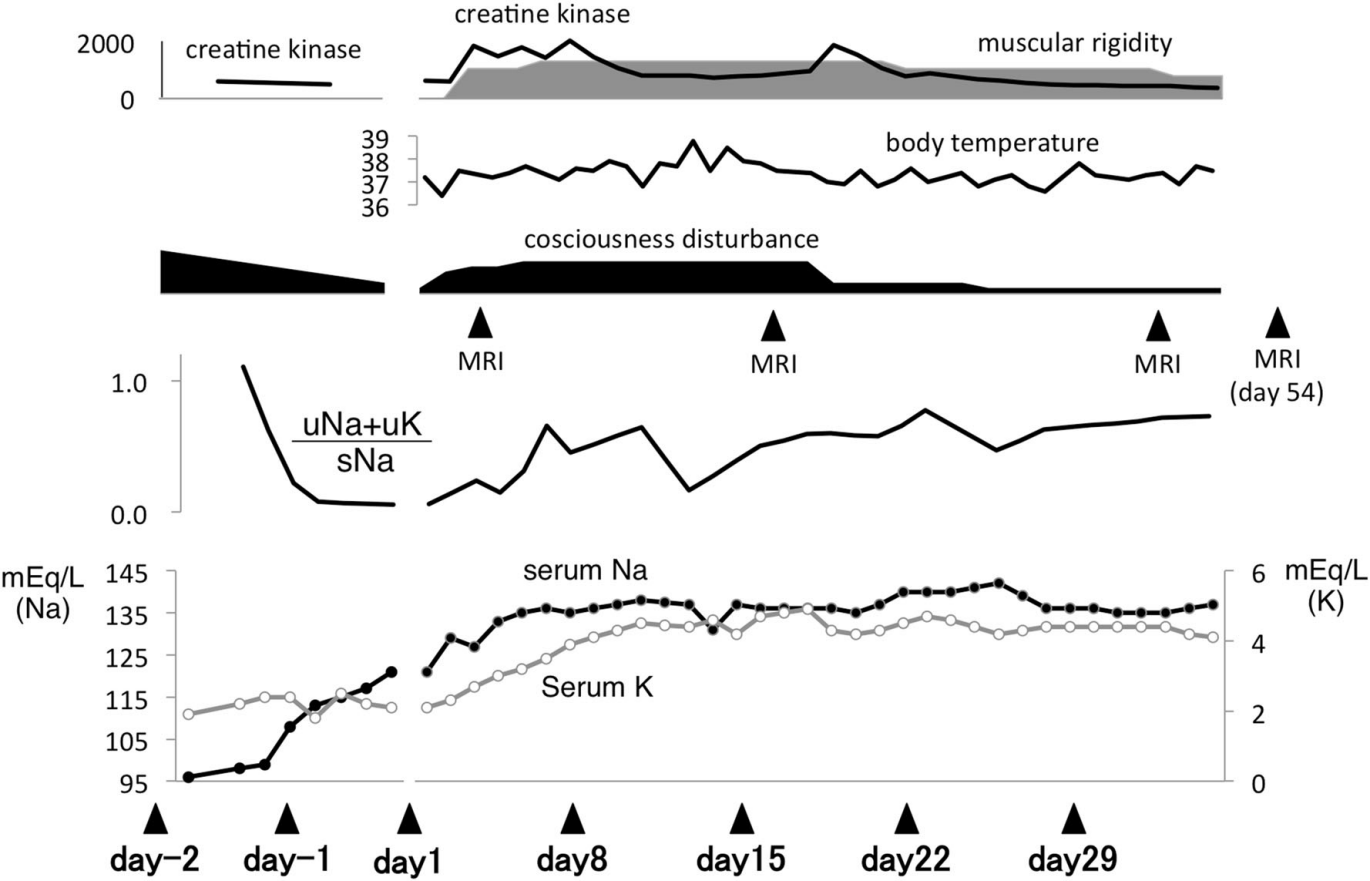
Clinical course of the present case, a 30-year-old Japanese male

In recent years, measurement of the serum-to-urine cation ratio [(urine Na + urine K)/serum Na] has been advocated as a method for easily assessing free water excretion [3]. If this value is ≥1.0, it suggests a state in which free water excretion may be suppressed, especially in a hyponatremic setting [1]. As shown in Fig. 2, the cation ratio of our patient was ≥1.0 at the time of his admission to the emergency hospital. However, after the discontinuation of the antipsychotic agents, a decrease in the cation ratio and a concomitant decrease in the serum Na concentration were observed.

Whether the early effects of discontinuation of the antipsychotic agents can take place as observed in the present case remains controversial, as we were unable to measure the patient’s ADH concentration. However, we believe that the impaired free water excretion could well be the main cause for the hyponatremia in this case. In addition, the patient had a history of consuming ≥10 L of water daily prior to admission, and although the details remain unclear, he did have a history of admission for water intoxication. We thus feel that it is highly likely that the patient had been in a state close to psychotic polydipsia, and this may have facilitated the development of severe hyponatremia.

The patient’s serum K concentration was 1.9 mEq/L at the time of admission to the emergency hospital. Because the fractional K excretion (FEK) was high, excess K excretion by the kidney may have had a strong influence. On his admission to the emergency hospital, our patient showed remarkable polyuria reaching 6 L/day, and his FEK level decreased in parallel with a decrease in urine volume. The possible involvement of a hypokalemic nephropathy-related urine concentration disturbance should be considered, but the patient’s polyuria was almost restored within the first 3 days before the normalization of his serum K concentration, suggesting that the massive water intake would be more likely as a cause of the polyuria.

It is known that K excretion generally increases together with an increase in urine flow through the renal collecting ducts [4], and we believe that we cannot rule out the possibility that the remarkable polyuria in the present case played a major role in the renal K excretion. Moreover, the alkalemia and metabolic alkalosis improved almost in parallel with the improvement in hypokalemia, and we thus believe that this was an acid–base imbalance associated with hypokalemia.

Our patient’s persistent consciousness disturbance is the next topic to be considered. The consciousness disturbance observed at the time of his admission to the emergency hospital is thought to have been caused by the severe hyponatremia, and improvement was observed as his serum Na concentration was corrected. However, despite improvement in the serum Na value, the consciousness disorder reappeared and persisted for a long time. We will first discuss the possibility of CPM or extrapontine myelinolysis (EPM).

The patient’s serum Na concentration was corrected to the rate 30 mmol/L within 3 days after admission to the emergency hospital, and thus, the Na correction rate might have been excessive. The guidelines on hyponatremia correction recently published by the European Renal Association state that serum Na should be corrected with 10 mmol/L during the first 24 h and within 8 mmol/L from the second day onwards, and moreover, that blood tests should be performed every 6 h during correction [5]. CPM is well known to be associated with an excessive correction of the serum Na concentration, but a disease concept was recently advocated that includes EPM and regards neurological symptoms arising from changes in osmotic pressure as an osmotic demyelination syndrome (ODS) [6].

Kallakatta et al. listed three risk factors for ODS onset on the basis of their analysis of 25 cases: (1) serum Na ≤115 mmol/L, (2) the presence of hypokalemia, and (3) higher GCS scores [7]. In the present case, the patient had a serum Na concentration of 96 mmol/L and a GCS score of 10 at the time of admission to the emergency hospital, as well as a serum K concentration of 1.9 mmol/L, which fulfills all of these criteria, and he was thus considered to be at high risk for the development of ODS. EPM image findings often demonstrate hypointense on T1W1 and hyperintense on T2W1 in the striatum, thalamus, geniculate body, and cerebellum [8]. In our patient, hyperintensity was also observed on T2-weighted imaging in the ventral lateral thalamic nucleus, which was consistent with an image finding of EPM. However, in the present case, the hyperintensity on T2-weighted images persisted after his consciousness disorder disappeared.

There are many cases in which abnormal findings on MRI scans disappeared after EPM/CPM symptoms disappeared [9], which is inconsistent with the present case. In addition, EPM generally presents with a variety of clinical symptoms [8], but there are no reports of the disorder presenting with a consciousness disturbance, to the best of our knowledge. There are case reports in which abnormal findings were observed in the thalamus on an MRI scan, and Parkinson-like symptoms, such as bradykinesia and tremor, were seen [10] or abnormal findings were observed in the basal ganglia, and disorientation and mutism were exhibited [11]. However, no consciousness disturbance was described in the above cases. Taking the above reports into consideration, it is difficult to explain our patient’s persistent disturbance of consciousness and muscular rigidity (the main abnormal neurological components) solely on the basis of thalamic abnormalities.

The consciousness disorder in the present case was associated with fever and muscular rigidity, and the patient was prescribed a number of antipsychotic agents for schizophrenia. Considering the fact that all antipsychotic agents were discontinued after admission to the emergency hospital, it was considered that malignant hyperthermia syndrome associated with the discontinuation of the antipsychotic agents is also a candidate for the differential diagnosis. Malignant hyperthermia syndrome presents with a precipitous fever, consciousness disturbance, extrapyramidal disorder, autonomic nervous system symptoms, and rhabdomyolysis are observed after an increase, change or discontinuation of antipsychotic agents [12, 13]. In addition, it is known that the amount of time elapsed from the discontinuation of antipsychotic agents until the onset of this syndrome appears to be within 24 h in 16 % of cases, within one week in 66 %, and within 30 days in 96 % [14].

In the present case, fever, a consciousness disturbance, muscular stiffness that was believed to represent extrapyramidal symptoms, and excessive sweating that was believed to represent autonomic nervous system symptoms occurred 4 days after the discontinuation of antipsychotic intake. We thus feel that the present patient fulfilled the diagnostic criteria of Levenson et al. [15]. However, it is difficult to explain his MRI findings by malignant hyperthermia syndrome alone. Accordingly, it appears appropriate to conclude that the present case represents complications of concomitant ODS and malignant hyperthermia syndrome; however, the consciousness disturbance itself can be explained by ODS.

In summary, we encountered a patient presenting with severe hyponatremia caused by drug-induced SIADH followed by a persistent consciousness disturbance and characteristic MRI findings that resulted from a concomitant onset of ODS and malignant hyperthermia syndrome. Dilutional hyponatremia associated with drug-induced SIADH is a highly recognized condition, and in such a case, the discontinuation of all antipsychotic agents is a generally acceptable clinical strategy. In such a clinical setting, the possible development of malignant hyperthermia syndrome should be considered.

## References

[1] Ohsawa H, Kishimoto T, Hirai M, Shimayoshi N, Matsumura K, Oribe H, Hirao F, Ikawa G, Nakai T, Miyake M (1992). An epidemiological study on hyponatremia in psychiatric patients in mental hospitals in Nara Prefecture. Jpn J Psychiatry Neurol..

[2] Manu P, Ray K, Rein JL, De Hert M, Kane JM, Correll CU (2012). Medical outcome of psychiatric inpatients with admission hyponatremia. Psychiatry Res.

[3] Adrogue HJ, Madias NE (2012). The challenge of hyponatremia. J Am Soc Nephrol.

[4] Wang WH, Giebisch G (2009). Regulation of potassium (K) handling in the renal collecting duct. Pflugers Arch.

[5] Spasovski G, Vanholder R, Allolio B, Annane D, Ball S, Bichet D, Decaux G, Fenske W, Hoorn EJ, Ichai C, Joannidis M, Soupart A, Zietse R, Haller M, van der Veer S, Van Biesen W, Nagler E (2014). Clinical practice guideline on diagnosis and treatment of hyponatraemia. Nephrol Dial Transpl.

[6] Kleinschmidt-Demasters BK, Rojiani AM, Filley CM (2006). Central and extrapontine myelinolysis: then…and now. J Neuropathol Exp Neurol.

[7] Kallakatta RN, Radhakrishnan A, Fayaz RK, Unnikrishnan JP, Kesavadas C, Sarma SP (2011). Clinical and functional outcome and factors predicting prognosis in osmotic demyelination syndrome (central pontine and/or extrapontine myelinolysis) in 25 patients. J Neurol Neurosurg Psychiatry.

[8] Brown WD (2000). Osmotic demyelination disorders: central pontine and extrapontine myelinolysis. Curr Opin Neurol.

[9] Yuh WT, Simonson TM, D’Alessandro MP, Smith KS, Hunsicker LG (1995). Temporal changes of MR findings in central pontine myelinolysis. AJNR Am J Neuroradiol.

[10] Tsai MH, Lu CS, Chen CJ, Tsai WP, Liou LB (2002). Extrapontine myelinolysis in a patient with systemic lupus erythematosus: a case report. J Formos Med Assoc.

[11] Salvesen R (1998). Extrapontine myelinolysis after surgical removal of a pituitary tumour. Acta Neurol Scand.

[12] Nelson TE, Flewellen EH (1983). Current concepts. The malignant hyperthermia syndrome. N Engl J Med.

[13] Strawn JR, Keck PE, Caroff SN (2007). Neuroleptic malignant syndrome. Am J Psychiatry.

[14] Caroff SN, Mann SC (1993). Neuroleptic malignant syndrome. Med Clin North Am.

[15] Levenson JL (1985). Neuroleptic malignant syndrome. Am J Psychiatry.

